# Evidence for a relationship between genetic polymorphisms of the L-DOPA transporter LAT2/4F2hc and risk of hypertension in the context of chronic kidney disease

**DOI:** 10.1186/s12920-024-01935-2

**Published:** 2024-06-18

**Authors:** Paolina Crocco, Serena Dato, Rossella La Grotta, Giuseppe Passarino, Giuseppina Rose

**Affiliations:** Department of Biology, Ecology and Earth Sciences, University of Calabria, 87036 Rende (CS), Italy

**Keywords:** Chronic kidney disease, Hypertension, L-DOPA, LAT2*/SLC7A8*, 4F2hc*/SLC3A2*, SNP

## Abstract

**Background:**

Chronic kidney disease (CKD) and hypertension are chronic diseases affecting a large portion of the population frequently coexistent and interdependent. The inability to produce/use adequate renal dopamine may contribute to the development of hypertension and renal dysfunction. The heterodimeric amino acid transporter LAT2/4F2hc (*SLC7A8/SLC3A2* genes) promotes the uptake of L-DOPA, the natural precursor of dopamine. We examined the plausibility that *SLC7A8/SLC3A2* gene polymorphisms may contribute to hypertensive CKD by affecting the L-DOPA uptake.

**Methods:**

421 subjects (203 men and 218 women, mean age of 78.9 ± 9.6 years) were recruited and divided in four groups according to presence/absence of CKD, defined as reduced estimated glomerular filtration rate (eGFR < 60 ml/min/m^2^) calculated using the creatinine-based Berlin Initiative Study–1 (BIS1) equation, and to presence/absence of hypertension (systolic blood pressure ≥ 140 and/or diastolic blood pressure ≥ 90 mmHg). Subjects were analysed for selected SNPs spanning the *SLC7A8* and *SLC3A2* loci by Sequenom MassARRAY iPLEX platform.

**Results:**

The most significant SNP at the *SLC3A2* (4F2hc) locus was rs2282477-T/C, with carriers of the C-allele having a lower chance to develop hypertension among CKD affected individuals [OR = 0.33 (CI 0.14–0.82); *p* = 0.016]. A similar association with hypertensive CKD was found for the *SLC7A8* (LAT2) rs3783436-T/C, whose C-allele resulted associated with decreased risk of hypertension among subjects affected by CKD [OR = 0.56 (95% CI 0.35–0.90; *p* = 0.017]. The two variants were predicted to be potentially functional.

**Conclusions:**

The association between *SLC3A2* and *SLC7A8* variants to hypertension development in patients with renal failure could be linked to changes in L-DOPA uptake and consequently dopamine synthesis. Although the associations do not survive correction for Bonferroni multiple testing, and additional research is needed, our study opens new avenues for future basic and translational research in the field of hypertensive CKD.

**Supplementary Information:**

The online version contains supplementary material available at 10.1186/s12920-024-01935-2.

## Background

The progressive decline of renal function that accompanies the physiological aging has received considerable attention as it is the leading risk factor for chronic kidney disease (CKD), a condition that can deteriorate further throughout life, potentially resulting in kidney failure or end-stage renal disease (ESRD) [1]. A measure of the kidney’s filtration ability widely used clinically to diagnose and stage CKD is decreased glomerular filtration rate (GFR), which can be estimated from the serum creatinine level (eGFRcrea). eGFR shows a strong heritable component, [2] and recent multi-ethnic genome-wide association studies (GWAS) and meta-analysis identified multiple genetic loci associated with CKD and eGFR, that can explain a portion, albeit, small, of the trait variance. [3, 4].

To date, a huge body of research has consistently found that loss of renal function is a strong risk factor for adverse clinical outcomes and increased mortality in the general population [5]. In particular, numerous evidence indicate that CKD is an important complicating factor for hypertension, a disorder that compromises cardiovascular health, and that is increasing in incidence and prevalence worldwide, becoming a major public problem [6]. The relationship between CKD and hypertension is reciprocal since hypertension is a strong determinant of worse renal function and decline in renal function frequently causes or exacerbates hypertension. This close interconnection is the result of multiple factors, including reduced nephron mass, increased sodium retention, endothelial dysfunction, and activation of hormones [7–9].

Dopamine is a natriuretic and diuretic hormone with an important paracrine/autocrine role in the kidney, affecting water and electrolyte balance. Increasing research indicate that the activation of intrarenal dopaminergic pathways, both for dopamine production and signalling, helps prevent or mitigate the development of hypertension, whereas defects in this system are linked to increased susceptibility to hypertension [10–12].

The major source of renal dopamine is derived from the decarboxylation of its precursor l-3, 4-dihydroxyphenylalanine (L-DOPA), which is taken up by proximal renal tubules from either the circulation or the glomerular filtrate and is then converted to dopamine by the aromatic L-amino acid decarboxylase (L-AADC), also localized to the renal tubules, the activity of which is upregulated by high-sodium diet and downregulated by low-salt diet [13, 14]. Cell uptake of L-DOPA, which may be the rate-limiting step in the synthesis of renal dopamine, is promoted mainly through the sodium independent and pH-sensitive L-type amino acid transporter type 2 (LAT2), which is present at both apical and basolateral membranes [15]. LAT2 is a non-glycosylated 12-transmembrane-spanning membrane protein, and a member of the solute carrier family 7 (SLC7) of amino acid transporters, coded by the *SLC7A8* gene. Functional expression of LAT2 requires association with the 4F2 heavy chain antigen (4F2hc), also known as CD98, to form a heterodimer [16]. 4F2hc belongs to the solute carrier family 3 member 2 and is coded by the *SLC3A2* gene. Consistent with a role of LAT2 in transporting L-DOPA, Wu et al. [17] demonstrated that by increasing the expression of *SLC7A8* in tubular epithelial cells a corresponding increase in L-DOPA uptake was achieved. LAT2 has been also reported to be expressed at higher levels in renal cortex of spontaneously hypertensive (SHR) than in normotensive rats; its overexpression was associated with enhanced L-DOPA uptake and preceded the onset of hypertension [18, 19].

The impairment of renal dopaminergic system functionality in hypertension has been extensively studied at various levels both in human and in experimental animals [11, 20]. However, to our knowledge, no studies to date have examined the effect of genetic variants of *SLC7A8* and *SLC3A2* genes on the relationship between CKD and hypertension. Therefore, the purpose of this exploratory study is to addresses this knowledge gap by investigating this effect in a sample population of elderly subjects.

## Methods

### Study participants

For this study we analysed 421 unrelated subjects (203 men and 218 women) whose ages ranged from 64 to 90 years. All subjects were born in Calabria (South Italy) and ancestry ascertained by requiring up to the grandparents' generation to be in the same region. Sample recruitment was undertaken across the whole territory through several recruitment campaigns carried out for monitoring the quality of aging in the whole Calabria region (21). The recruitment strategies consisted in contacting the family physicians and nursing home and examining population registers. Subjects who were eligible for the study were contacted and invited to participate in the study. Each subject underwent a medical visit carried out by a geriatrician who also conducted an interview including the administration of a structured questionnaire. The questionnaire collected socio-demographic information, evaluated physical and cognitive status, and self-reported health status. Subjects with cancer, dementia and/or neurologic disorders were not included. Before the visit, an informed consent was signed by each subject for the permission to collect blood samples and usage of register-based information for research purposes. The recruitment flow chart of the study participants is reported in the Additional file 1.

### Biochemical measurements

At the time of enrolment, a peripheral blood sample was drawn after an overnight fast of 12 h in the morning for clinical and laboratory screening. Serum samples were immediately stored at -80 °C until assayed. The general laboratory panel included serum creatinine measured using the standardized Jaffe method calibrated to isotope dilution mass spectrometry using the automated analyser (RX-30, Nihon Denshi Inc., Tokyo, Japan).

### Estimated Glomerular Filtration Rate Equation and blood pressure measurement

The GFR was estimated by the creatinine-based Berlin Initiative Study – 1 (BIS1) equation specially used to estimate GFR in older adults [22]. The equation was the following: eGFR BIS1 = 3736 × creatinine^−0.87^ × age^−0.95^ × 0.82 (if female).

Blood pressure was measured by a trained physician using a standard mercury sphygmomanometer. All measurements were performed three times after at least 5 min of rest from the left arm in a sitting posture.

### Definition of study phenotypes

We defined a participant with CKD as an individual with eGFR < 60 mL/min/1.73m^2^ based on the BIS1 equation, and a participant with hypertension as an individual with elevated systolic blood pressure (SBP) or diastolic blood pressure (DBP) (SBP/DBP ≥ 140/90 mm Hg or taking antihypertensive medications). According to these criteria, study participants were categorised into one of four groups: [G1] healthy individual without CKD and hypertension (CKD-/Hyper-); [G2] individual without CKD and with hypertension (CKD-/Hyper +); [G3] individual with CKD and without hypertension (CKD + /Hyper-); [G4] individual with CKD and hypertension (CKD + /Hyper +).

### Single nucleotide polymorphisms (SNPs) selection

SNPs within the *SLC7A8* and *SLC3A2* gens were selected based on a tagging approach to cover most of the genetic variability of these genes, using the Haploview Tagger Program software (https://www.broadinstitute.org/haploview/haploview, version 4.2). The criteria for the selection of tagging SNPs were minor allele frequency (MAF) higher than 0.05 and r^2^ higher than 0.8. A total of fifteen SNPs were selected for genotyping, eight of which were in the *SLC7A8* gene and seven in the *SLC3A2* genes. For each SNP, information related to the chromosome, position, minor allele frequency (MAF) detected in the control subjects of this study and in the 1000 Genomes TSI (Tuscany, Italy) population, and functional annotation of the SNP (missense, synonymous, intronic, and noncoding) is given in Additional file 2.

### DNA extraction and genotyping

Genomic DNA was isolated from whole blood or buffy coats according to the salting out procedure.

Multiplex Genotyping was performed using the iPLEX assay on MassArray system (Sequenom Inc., San Diego, CA, USA). Sequenom MassARRAY Assay Designer software (version 3) was used to design primers for PCR and for single base extension against the candidate SNPs selected from analysis. PCR products were processed following the manufacturer’s instructions, and unincorporated nucleotides deactivated with the shrimp alkaline phosphatase (SAP). A primer extension reaction was then used to generate allele-specific products, which were subsequently desalted on resin and spotted onto the 384-element SpectroCHIP (Sequenom) for matrix-assisted laser desorption/ionization-time of flight (MALDI-TOF) mass spectrometry analysis using SpectroACQUIRE v3.3.1.3 (Sequenom). The MassARRAY Typer v3.4 software (Sequenom). was used to call genotypes, and the individual spectrograms were inspected to check for calling errors.

To assess the reliability of the genotype identification protocols, about 10% of the analyzed samples were re-genotyped. For all SNPs polymorphisms concordance among duplicates was about 100%.

### Quality Control

After genotyping, samples were subjected to a battery of quality control (QC) tests. At sample level, subjects with a proportion of missing genotypes higher than 10% were excluded from the analysis. At SNP level, SNPs were excluded if they had a missing frequency (MiF) higher than 20%, a minor allele frequency (MAF) lower than 5%, or a significant deviation from Hardy–Weinberg equilibrium (HWE, p < 0.05).

### Statistical analysis

Continuous variables are expressed as mean ± SDs while categorical variables as percentages. Kolmogrov-Simirnov and Shapiro–Wilk tests were used to evaluate the normal distribution of the variables. One-way Analysis of Variance (ANOVA) with the Bonferroni post-hoc test, and chi-square test were applied to assess differences in biochemical, clinical, and anthropometric variables among groups, where appropriate. Allele and genotype frequencies were calculated by direct counting from the observed genotypes. HWE was tested by Fisher’s exact test. Odd ratios (OR) and 95% confidence interval (95% CI) based on logistic regression models were estimated to evaluate the effect of genotypes (independent variables) on the probability of belonging to different groups (dependent variable) including age and sex as covariates. For each SNP, data were coded with respect to a dominant, a recessive, and an additive model of inheritance. The dominant model compares homozygous carriers for the less common allele and heterozygous subjects (grouped together) with the group of homozygous for the more common allele. The recessive model compares individuals homozygous for the minor allele with the combined group of heterozygous and major allele homozygous carriers. The additive model assumes that there is a linear gradient in risk between the three genotypes. The most likely genetic model was estimated based on minimum level of statistical significance (Wald test p-value).

A conditional analysis was performed by including associated SNPs in the model to test their independent effect.

All statistical data were analyzed by the SPSS software version 28.0 (SPSS, Inc., Chicago, IL, USA). The significance level of the association test was set at 5%. Bonferroni correction was performed to correct for multiple comparisons, setting the significant P-value at 2E-04 for the genetic analyses (0.05/13*3*6, where 13 represents the number of SNPs analyzed, 3 represents the genetic models and 6 the number of comparisons as reported in Table 2.

### Functional characterization of the single nucleotide polymorphisms

Several tools were used to investigate the functional effect of the associated SNPs. HaploReg v4.2 (https://pubs.broadinstitute.org/mammals/haploreg/haploreg.php) and RegulomeDB 2.2 (http://regulome.stanford.edu/) were used to investigate the potential influence of the SNPs on DNA regulatory elements. Specifically, HaploReg provides data in relation to the effect of the SNPs on chromatin state and regulatory motifs, while RegulomeDB provides a predictive score which reflect the probability for the SNP to be functionally active, and a rank score that depends on the amount of experimental evidence supporting the SNP to be functionally active. The rank ranges from 1 to 6 with a low score representing strong evidence of regulatory function. Genotype-Tissue Expression (GTEx) portal (https://gtexportal.org/home/) was interrogated to identify potential expression quantitative trait loci (eQTLs), which are genetic variants with an effect on gene expression levels.

## Results

### Characteristics of study subjects

The study population consisted of 421 subjects with a sex ratio of approximately 1:1 and a mean age of 78.9 ± 9.6 years. Overall, 59.14% had chronic kidney disease (CKD), defined by low eGFR (< 60 ml/min/m^2^), while 56.77% had hypertension, defined as systolic blood pressure ≥ 140 and/or diastolic blood pressure ≥ 90 mmHg. We divided the subjects into four phenotypic groups according to the presence/absence of CKD and/or hypertension. Group 1 included healthy individuals with no CKD and no hypertension (G1, CKD-/hyper-), group 2 persons without CKD with hypertension (G2, CKD-/hyper +), group 3 included persons with CKD without hypertension (G3, CKD + /hyper-), and group 4 persons with CKD and hypertension (G4, CKD + /hyper +).

The main demographic, clinical and biochemical characteristics of the whole sample are presented in Additional file 3, while Table 1 reports the characteristics of the four groups studied and the results of the comparisons among the four phenotypic groups.

**Table 1:** Clinical characteristics of the study participants categorized into four phenotype groups

Groups	G1	G2	G3	G4	
	CKD-Hyper- (*n* = 83)	CKD-Hyper + (*n* = 89)	CKD + Hyper- (*n* = 99)	CKD + Hyper + (*n* = 150)	*P*-value*
Age, years	72 (7.01)	71.63 (4.9)	82.9 (8.45) ^1,2^	83.27 (8.9) ^1,2^	** < 0.001**
Gender, males %	61.4	46.1^1^	46.5^1^	43.3^1^	** < 0.001**
Body mass index, kg/m^2^	26.08 (4.02)	26.21 (3.91)	25.09 (3.78)	26.41 (4.76)	ns
Systolic blood pressure, mmHg	129.38 (15.84)	138.17 (17.48)^1,3^	130.94 (11.93)	140.40 (14.60)^1,3^	** < 0.001**
Diastolic blood pressure, mmHg	73.79 (8.10)	77.83 (10.17)^1^	75.77 (7.20)	77.41 (8.23)^1^	**0.025**
Triglycerides, mg/dl	111.19 (44.92)	133.28 (62.81)	130.38 (81.06)	143.75 (77.87)^1^	**0.025**
HDL-cholesterol, mg/dL	56.67 (13.88)	55.31 (10.83)	56.22 (15.02)	54.48 (14.77)	ns
LDL cholesterol, mg/dl	124.68 (34.95)	123.24 (35.18)	121.73 (38.49)	123.23 (33.10)	ns
Total cholesterol, mg/dl	203.60 (41.08)	205.20 (38.80)	203.57 (47.37)	206.25 (41.75)	ns
Fasting plasma glucose, mg/dL	107.13 (37.86)	108.79 (23.71)	103.78 (31.93)	113.41 (43.58)	ns
Glycated hemoglobin, %	5.55 (0.97)	5.63 (0.77)	5.56 (0.88)	5.60 (1.18)	ns
Albumin (g/dL)	3.90 (0.32)	3.91 (0.28)	3.90 (0.35)	3.89 (0.32)	ns
Total protein (g/dL)	6.78 (0.45)	6.94 (0.43)	6.93 (0.56)	6.91 (0.50)	ns
Creatinine (mg/dL)	0.85 (0.15)	0.85 (0.14)	1.18 (0.46)^1,2^	1.21 (0.35)^1,2^	**< 0.001**
Urea (mg/dL)	31.50 (7.24)	33.04 (7.91)	44.00 (22.23)^1,2^	43.53 (16.05)^1,2^	**< 0.001**
Uric acid (mg/dL)	4.42 (1.16)	4.86 (1.10)	5.07 (1.56) ^1^	5.61 (1.62)^1,2,3^	**< 0.001**
Sodium (mM/L)	139.94 (2.35)	139.9 (2.43)	139.03 (2.35)	139.06 (2.56)	ns
Potassium (mM/L)	4.27 (0.39)	4.33 (0.37)	4.43 (0.44)	4.43 (0.48)	ns
Chloride (mM/L)	104.71 (2.30)	104.14 (2.26)	104.47(2.56)	103.24 (2.54)	ns
Calcium (mg/dL)	9.37 (0.38)	9.32 (0.39)	9.16 (0.72)	9.32 (0.54)	ns
Phosphorus (mg/dL)	3.44 (0.53)	3.61 (0.94)	3.44(0.74)	3.36 (0.62)	ns
Magnesium (mg/dL)	2.01 (0.26)	1.95 (0.29)	2.04 (0.28)	2.03 (0.29)	ns
Iron (μg/dL)	83.27 (46.44)	76.54 (33.31)	83.80 (426.55)	78.39 (32.57)	ns
Ferritin (ng/mL)	106.30 (89.74)	92.92 (96.77)	129.96 (156.39)	141.39 (216.44)	ns
Total biluribin (mg/dL)	0.70 (0.25)	0.78 (0.34)	0.77 (0.45)	0.70 (0.29)	ns
Alkaline phosphatase (U/L)	96.40 (55.92)	89.19 (29.12)	112.03 (68.38)	104.81(79.59)	ns
C-Reactive Protein (mg/L)	5.33 (9.25)	4.71 (5.93)	7.26 (12.08)	6.28 (12.96)	ns

Variables are expressed as mean (standard deviation). *P value from ANOVA for continuous variables and from chi-squared tests of association for categorical variables. ^1^*p* < 0.05 vs. G1 [CKD-Hyper-], ^2^*p* < 0.05 vs. G2 [CKD-Hyper +], ^3^*p* < 0.05 vs. G3 [CKD + Hyper-], ^4^*p* < 0.05 vs. g4 [CKD + Hyper +] from Bonferroni multiple comparison test

With respect to individuals without CKD, both with and without hypertension, those with CKD, both with and without hypertension, were older. Also, there was a lower proportion of males in all groups compared to the proportion of males in the control (G1) group. Moreover, significant differences among groups in some disease-related quantitative metabolic variables were observed. As shown in Table 1 and as expected, the means for both systolic and diastolic blood pressure were found significantly higher in subjects with hypertension both with and without CKD, when compared with normotensive subjects. On the other hand, higher levels of creatinine, urea and uric acid were found among subjects with CKD, both hypertensive and normotensive when compared with the other groups. Finally, compared with the control group CKD + /hyper + subjects showed higher levels of triglycerides.

### Genetic association of LAT2 and 4F2hc SNPs to chronic kidney disease and hypertension

The complete list of the selected SNPs is reported in Additional file 2. No deviations from HWE among the controls were observed, except for rs489381 (*SLC3A2*), rs1015089 (*SLC7A8*). Therefore, these two SNPs were excluded from further analysis.

The potential implication of the selected variants on the probability of belonging to a specific phenotypic group was tested by logistic regression analyses comparing each group with every other group. Results are summarized in Table 2.

**Table 2:** Results of the association tests for the selected SNPs, resulting from the logistic regression analysis

	G1 vs G2		G1 vs G3		G1 vs G4		G2 vs G3		G2 vs G4		G3 vs G4	
SNP	CKD-/Hyper- *vs* CKD-/Hyper +		CKD-/Hyper- *vs* CKD + /Hyper-		CKD-/Hyper- *vs* CKD + /Hyper +		CKD-/Hyper + *vs* CKD + /Hyper-		CKD-/Hyper + *vs* CKD + /Hyper +		CKD + /Hyper- *vs* CKD + /Hyper +	
	OR (95% CI)	*P*	OR (95% CI)	*p*	OR (95% CI)	*p*	OR (95% CI)	*P*	OR (95% CI)	*P*	OR (95% CI)	*P*
***SLC7A8/LAT2***												
rs72684330-T/A	2.47 (0.61–9.93)	0.20	1.37 (0.35–5.27)	0.65	1.99 (0.59–6.74)	0.27	1.01 (0.32–3.21)	0.98	1.51 80.56–4.05)	0.41	1.15 (0.54–2.46)	0.703
rs17794251-C/T	0.60 (0.27–1.34)	0.21	0.95 (0.38–2.36)	0.91	0.84 (0.38–1.85)	0.66	1.17 (0.48–2.85)	0.73	1.17(0.56–2.46)	0.67	1,04 (5.83–1.85)	0.89
** rs3783436 T/C**	0.96 (0.42–2.25)	0.93	1.63 (0.59–4.49)	0.34	0.81 (0.35–1.89)	0.63	1.96 (0.95–4.02)	0.066	0.89 (0.42–1.93)	0.78	**0.56 (0.35–0.90)**	**0.017**^**A**^
rs999165 T/A	0.83 (0.34–2.05)	0.69	1.39 (0.54–3.58)	0.49	1.63 (0.68–3.91)	0.27	1.53 (0.62–3.81)	0.35	1.83 (0.79–4.19)	0.15	1.28 (0.71–2.31)	0.40
rs12588118 C/G	1.02 (4.41–2.26)	o.96	1.22 (0.48–3.09)	0.67	0.81 (0.35–1.84)	0.61	0.96 (0.39–2.35)	0.92	0.64 (0.29–1.36)	0.25	0.67 (0.38–1.20)	0.18
rs10150592 C/A	0.89 (0.38–2.12)	0.80	0.58 (0.22–1.53)	0.27	0.67 (2.77–1.60)	0.36	0.65 (0.26–1.60)	0.35	0.81 (0.36–1.80)	0.59	0.79 (0.43–1.47)	0.47
rs7141505 C/A	1.44 (0.64–3.26)	0.38	1.38 (0.53–3.58)	0.50	1.12 (0.49–2.50 =	0.79	0.71 (0.28–1.8)	0.47	o.61 (0.28–1.32)	0.21	0.94 (0.53–1.68)	0.84
***SLC3A2/CD98***												
rs12794763 T/G	0.48 (0.19–1-16)	0.11	0.24 (0.18–1.15)	0.14	0.71 (0.29–1.22)	0.43	0.53 (0.17–1.8)	0.28	1.49 80.64–3.51)	0.35	1.89 (0.82–4.36)	0.13
rs12221878 C/G	1.39 (0.48–4.02)	0.54	1.94 (0.53–7.12)	0.32	1.02 (0.33–3.18)	0.97	1.64 (0.51–5.27)	0.40	0.81 (0.33–1.98)	0.64	0.73 (0.35–1.55)	0.41
rs10792362 T/C	0.73 (0.27–1.99)	0.54	0.62 (0.21–1.7)	0.37	0.41 (0.16–1.04)	0.06	0.39 (1.55–4.28)	0.39	0.61 (0.27–1.37)	0.23	0.72 (0.38-.34)	0.31
rs12804553 G/T	0.76 (0.37–1.48)	0.47	1.20 (0.51–2.84)	0.67	0.94 (0.44–2.01)	0.88	1.63 (0.71–3.75)	0.24	1.22 (0.60–2.48)	0.57	0.99 (0.57–1.729	0.98
rs4726 C/T	0.91 (0.42–1.98)	0.81	0.47 (0.17–1.26)	0.14	0.95 (0.41–2.18)	0.91	0.39 (0.15–1.06)	0.066	0.94 (0.45–1.95)	0.87	1.49 (0.82–2.72)	0.19
** rs2282477 T/C**	0.49 (0.14–1.73)	0.27	0.60 (0.18–1.2)	0.40	**0.27 (0.18–0.91)**	**0.035**^**D**^	1.54 (0.42–5.66)	0.51	0.77 (0.21–2.27)	0.69	**0.33 (0.14–0.82)**	**0.016**^**D**^

After adjustments for age and sex, the most significant result at the SLC3A2/4F2hc locus was found at rs2282477-T/C. In particular, by comparing group G1 to group G4, that is the comparison between subjects with no CKD and no hypertension and subjects with CKD and hypertension, we observed a dominant genetic effect of rs2282477-C allele on the chance to belong to the G4 group with an odds ratio (OR) of 0.27 (CI 0.18–0.91; *p* = 0.035), meaning that that the presence of the minor allele C associates with reduced chance to have both CKD and hypertension in comparison to healthy controls. For rs2282477 in SLC3A2/4F2hc we also found association in a dominant model when comparing group G3 to group G4, that is the comparison between CKD subjects without versus those with hypertension, [OR = 0.33 (CI 0.14–0.82); *p* = 0.016]. This means that among CKD affected individuals, those carrying the rs2282477-C allele have a lower chance to develop hypertension relative to homozygotes for the T allele. A similar result was found for the marker of *SLC7A8*/LAT2 rs3783436-T/C, whose minor allele C resulted associated with reduced risk of hypertension when the G3 group was compared to the G4 in an additive model [OR = 0.56 (95% CI 0.35–0.90; *p* = 0.017]. It should be noted, however, that these associations did not meet the Bonferroni correction threshold for multiple testing (p_corrected_ = 0.0002). No associations were identified in the other comparisons.

To test whether the two SNPs (rs2282477 and rs3783436) have independent effect when considered together, we performed a conditional logistic regression analysis of each SNP with the other SNP as covariate and adjusting for age and sex. This analysis showed that the significant association of rs3783436 with the chance to belong to the G4 phenotype (comparison G3 versus G4) disappeared (p > 0.1) after adjusting for rs2282477, which is consistent with a non-independent effect of the two SNPs.

### Possible functional effects

The rs2282477 lies in the 3' untranslated region of 4F2hc gene while rs3783436 is located in intron 4 of LAT2 gene, so we checked whether there could be differences in gene expression due to these polymorphisms, to provide a rationale for the association found. To this aim, we used several online resources. First, we queried the Haploreg database for the presence of proxy SNPs in linkage disequilibrium (LD) (r^2^ >  = 0.8) with these variants. Rs2282477 resulted not in LD with any other polymorphisms, while rs3783436 was in LD with 5 intronic SNPs in the same gene. Further analysis of these SNPs using Haploreg indicated that they are in regions of active chromatin, as indicated by a DNASE I hypersensitivity site. Data from RegulomeDB support these observations, as both SNPs showed a score of 1f, which indicates they are affecting different DNA regulatory elements and are linked to the expression of target genes. To gain more insight in the possible regulatory roles of rs2282477 and rs3783436 on gene expression, Genotype-Tissue Expression project database (http://www.gtexportal.org/home/) was used. For both variants, no eQTL effect in renal tissue is reported in GTEx portal. However, both SNPs were expression quantitative trait (eQTL) and splicing quantitative trait (sQTL) loci in multiple tissues and cell types. Rs2282477 was an eQTL for *TMEM223* (Transmembrane Protein 223) gene in blood with a decreasing expression effect of the minor allele C. This variant was additionally demonstrated to represent a sQTL for *SLC3A2* in several cultured cell lines with the C allele associated with significantly decreased intron excision ratios. As for the rs3783436, we found that the less common C allele increased the expression of *SLC7A8* in several brain regions, and also resulted a sQTL of *SLC7A8* with tissue-specific splicing effects of the genotypes.

## Discussion

Chronic kidney disease (CKD) and hypertension are two common chronic diseases that often coexist and can have a bidirectional cause-effect relationship. Their coexistence has serious implications for human health increasing morbidity and mortality, especially from cardiovascular disease [23]. Several factors contribute to the concurrent presence of hypertension and kidney disease [24], including the contribution of a genetic component, as suggested by recent studies highlighting genes involved in both hypertension and kidney disease [25, 26]. In the present study, to increase the understanding of the genetic connection in between, we investigated the genetic variability of the *SLC7A8/SLC3A2* genes, coding for the heteromeric transporter LAT2/4F2hc which mediates the cell-uptake of L-DOPA in kidney, in subjects stratified according to presence/absence of CKD and presence/absence of hypertension. CKD was evaluated by estimated glomerular filtration rate (eGFR) according to the BIS1 equation, the most reliable for assessing renal function in older white patients and reported to perform relatively better than other equations in older adults [22, 27, 28].

Although not holding Bonferroni correction for multiple testing, our results provided suggestive evidence,, that the minor alleles of two variants, rs2282477-C within *SLC3A2*/4F2hc- and rs3783436-C within SC7A8/LAT2, were nominally associated with lower odds of hypertension in presence of CKD. The two SNPs seems to have no independent effect in reducing the risk of hypertension, most likely reflecting the functional linkage of the two genes.

Both SNPs lie in non-coding regions, specifically rs2282477 in the 3'-UTR and rs3783436 in intron 4 of the respective genes, thus having no obvious direct effect on the structure and function of the affected proteins. The *in-silico* analysis showed that these variants could alter the binding ability of some specific transcription factors that could alter gene expression. According to the GTEx data, both polymorphisms act as eQTL and sQTL with different genotype effects across different cell types/tissues. In particular, the *SLC3A2*-rs2282477 was identified as a cis-eQTL for the gene *TMEM223* in blood, with homozygous subjects for the protective C allele showing lower gene expression levels. *TMEM223* encodes for an inner mitochondrial membrane protein involved in the assembly process of the mitochondrial- encoded COX-1 and required for the biogenesis of cytochrome c oxidase (complex IV) [29].

As for the rs3783436-C allele, eQTL analysis found it associated with increased expression of *SLC7A8* mRNA in several brain regions. This is quite interesting considering that the brain and kidney act in concert to maintain normal body homeostasis by controlling sodium and water balance [30]. Unfortunately, no eQTL data from kidney tissue were available for these variants or their proxies in linkage disequilibrium. Nevertheless, based on available data, a plausible explanation for our results could be that an increase in LAT2 and/or 4F2hc gene expression owing to the presence of these genetic variants determines an increase of the uptake of L-DOPA and consequently of dopamine synthesis, which may lead decreased systemic blood pressure. This hypothesis is supported by Jacinto et al. [31], who reported reduced urinary dopamine/sodium excretion in some forms of human primary hypertension. Furthermore, hypertension has been reported in mice with intrarenal deletion of dopamine due to the aromatic L-amino acid decarboxylase (AADC) deficiency [32] or lacking dopamine receptors [33]. On the other hand, it has been reported that the expression of *SLC7A8* is increased in renal tissue of spontaneously hypertensive rats [17–19], which might contribute to enhanced L-DOPA uptake and increased dopamine production. It is tempting to speculate that in this setting the increased expression of *SLC7A8* may represent an adaptive compensatory mechanism, possibly caused by the deficiency in dopamine-mediated natriuresis, tending to limit the progressive rise blood pressure.

It is difficult, however, to establish a clear correlation between dopamine levels, LAT2/4F2hc expression, and the risk of hypertension in the context of CKD, even in view of the pleiotropic effects that dopamine and the SLC7A8/SLC3A2 genes exert. In fact, besides its important role in in helping to regulate blood pressure, dopamine enhances renal blood flow, an important determinant of glomerular filtration rate; low-dose dopamine represents a therapeutic choice to increase renal blood flow in patients, thus limiting or preventing renal failure [11]. Evidence shows that dopamine has also anti-inflammatory and antioxidant potential to prevent or ameliorate renal dysfunction [10, 11]. Mice with intrarenal dopamine deficiency have increased oxidative stress and inflammatory cells infiltration [32]. Furthermore, recent evidence has highlighted a relationship between the renal dopaminergic system and oxidative stress in the development of hypertension [20]. To this regard, it is worthwhile mentioning that rs2282477 SNP in *SLC3A2* acts as a cis-eQTL to modulate the expression of the *TMEM223* gene, which is involved in complex IV biogenesis whose dysfunction often leads to increased production of reactive oxygen species [29]. This suggests a plausible biological basis for the relationship among dopamine, oxidative stress, hypertension, and renal failure.

It is also worth noting that the LAT2/4F2hc complex, in addition to transport L-DOPA, also mediates the transport and reabsorption in proximal tubules of almost all neutral amino acids. Dysfunctions of this process may impact on maintaining amino acids concentration both in kidney and plasma [34]. This is of relevance considering the documented casual association of circulating amino acids with hypertension [35, 36] and with alteration in kidney function [37, 38]. For instance, glutamine, one of the amino acids transported by the LAT2/4F2hc complex, has been reported to significantly decrease kidney damage and improve kidney function by modulating oxidative stress and apoptosis in murine tubular epithelial cells [39].

We are aware that our work presents some limitations that merit consideration. First, the significance of our results did not survive correction for multiple testing. This is likely because the sample size within the subgroups is rather small, limiting statistical power to detect associations with likely moderate effects of SNPs. Since this is an exploratory study, findings must be interpreted with caution. Nevertheless, the results will be helpful for future confirmatory research conducted with larger population samples. A limitation is also that the consequences of these variants on gene expression in kidney is unknown and, hence, the biological mechanisms hypothesized to drive the associations are speculative. Further experimental studies are required to validate the findings. Lastly, considering that CKD includes various renal diseases, for future work, it would be interesting to consider whether the relationships we found differ according to specific kidney disorders.

## Conclusions

In conclusion, our preliminary findings derived from a hypothesis-driven candidate gene study offers suggestive evidence to support the role of *SLC3A2* /*SLC7A8* function to the development of hypertension in patients with renal failure. This association most probably reflect important pleiotropic effects of both dopamine and *SLC7A8/SLC3A2* genes. Given the prevalence and rising incidence rates of hypertensive CKD, understanding of pathogenic mechanisms and of potential determinants of these two associated conditions should be of utmost importance. Therefore, despite some limitations, we believe that our study has the important value of opening a novel avenue for future basic and translational research in the field of hypertensive CKD.

### Supplementary Information

Supplementary Material 1

Supplementary Material 2

Supplementary Material 3

Supplementary Material 4

## Data Availability

The dataset supporting the conclusions of this article is included in Additional file 4.

## Abbreviations

*4F2hc*: 4F2 heavy chain antigen
*AADC*: Aromatic L-amino acid decarboxylase
*BIS1*: Berlin Initiative Study–1
*CD98*: CD98 heavy chain
*CKD*: Chronic kidney disease
*COX-1*: Cytochrome c oxidase I
*DBP*: Diastolic blood pressure
*eGFR*: Estimated glomerular filtration rate
*eGFRcrea*: Estimated glomerular filtration rate from creatinine
*eQTLs*: Expression quantitative trait loci
*ESRD*: End-stage renal disease
*GTEx*: Genotype-Tissue Expression
*GWAS*: Genome-wide association studies
*HWE*: Hardy–Weinberg equilibrium
*L-AADC*: L-Amino Acid Decarboxylase)
*LAT2*: L-type amino acid transporter 2
*LD*: Linkage disequilibrium
*L-DOPA*: L-3, 4-dihydroxyphenylalanine
*MAF*: Minor allele frequency
*MALDI-TOF*: Matrix-assisted laser desorption/ionization-time of flight
*MiF*: Missing frequency
*PCR*: Polymerase chain reaction
*QC*: Quality control
*SBP*: Systolic blood pressure
*SHR*: Spontaneously hypertensive rats
*SLC3A2*: Solute carrier family 3 member 2
*SLC7A8*: Solute Carrier Family 7 Member 8
*SNPs*: Single nucleotide polymorphisms
*sQTL*: Splicing quantitative trait
*TMEM223*: Transmembrane Protein 223
*TSI*: Tuscany Italy
*WKY*: Wistar-Kyoto rats
