# Supplementary figures and images for: Evidence for a relationship between genetic polymorphisms of the L-DOPA transporter LAT2/4F2hc and risk of hypertension in the context of chronic kidney disease
Source: BMC Med Genomics. 2024 Jun 18;17:163. doi: 10.1186/s12920-024-01935-2 (PMC11186288; doi:10.1186/s12920-024-01935-2)

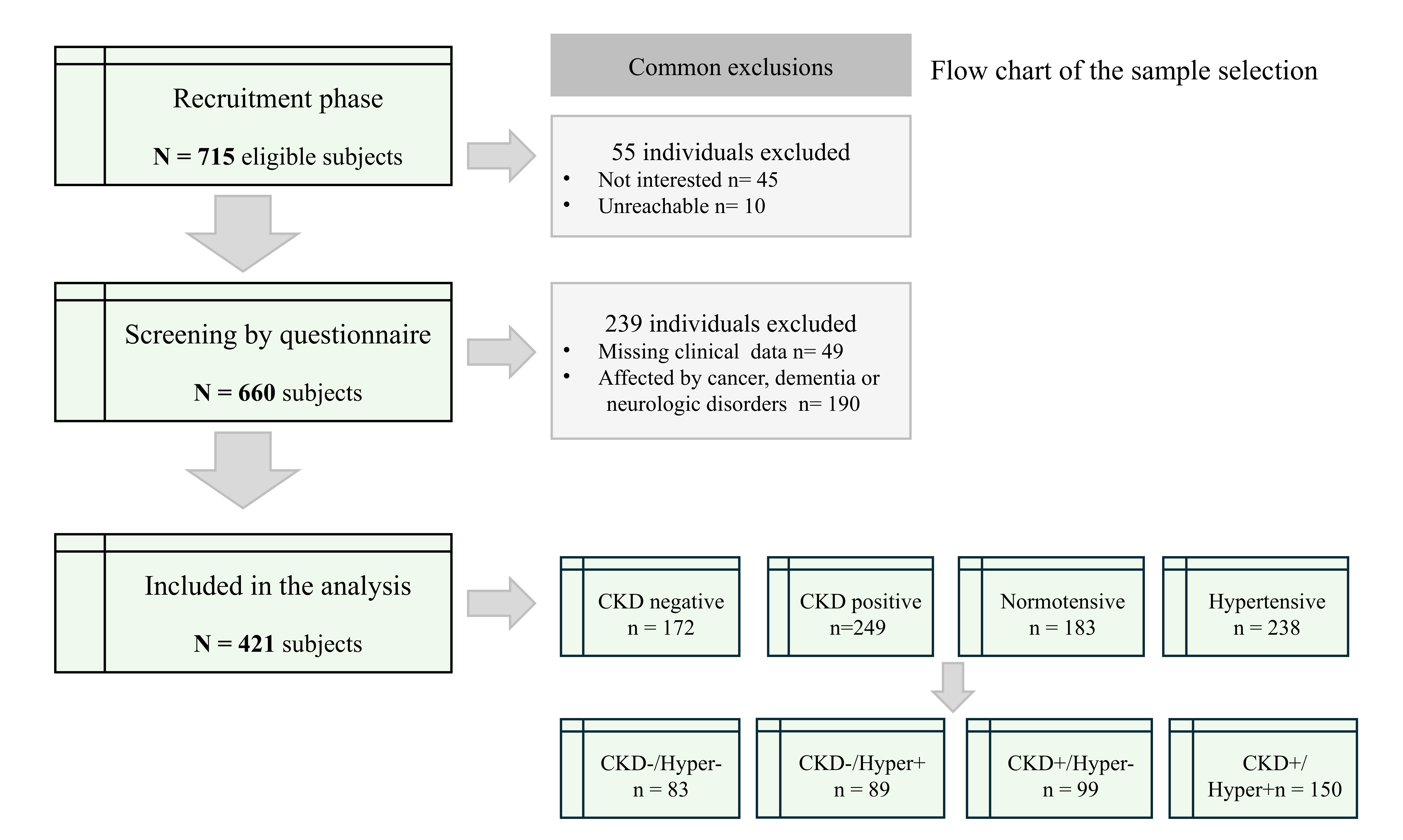

Supplement: Supplementary file 1 — Supplementary Material 1 [file 12920_2024_1935_MOESM1_ESM.tiff]
