# Supplementary material for: Evidence for a relationship between genetic polymorphisms of the L-DOPA transporter LAT2/4F2hc and risk of hypertension in the context of chronic kidney disease
Source: BMC Med Genomics. 2024 Jun 18;17:163. doi: 10.1186/s12920-024-01935-2 (PMC11186288; doi:10.1186/s12920-024-01935-2)
Supplement: Supplementary file 2 — Supplementary Material 2 [file 12920_2024_1935_MOESM2_ESM.docx]

**Additional file 1.** Description of the selected SNPs

| **SNP ID** | **Position** | **Functional annotation** | **MAF in controls** | **MAF in TSI from 1000 Genomes** |  |
| --- | --- | --- | --- | --- | --- |
| ***SLC7A8* (LAT2) Chr: 14** | | | | | |
| rs72684330 T/A | 23124197 | 3'near gene variant | 0.09 | 0.12 |  |
| rs17794251 C/T | 23124233 | 3'near gene variant | 0.35 | 0.27 |  |
| rs3783436 T/C | 23136764 | Intron variant | 0.34 | 0.34 |  |
| [rs1015089](https://www.ensembl.org/Homo_sapiens/Variation/Explore?db=core;g=ENSG00000092068;r=14:23125295-23183674;v=rs1015089;vdb=variation;vf=179670958) C/A | 23138037 | Intron variant | No HWE | 0.40 |  |
| rs999165 T/A | 23138657 | Intron variant | 0.22 | 0.27 |  |
| rs12588118 C/G | 23147985 | Intron variant | 0.30 | 0.23 |  |
| rs10150592 C/A | 23162327 | Intron variant | 0.23 | 0.17 |  |
| rs7141505 C/A | 23183979 | 5’near gene variant | 0.39 | 0.29 |  |
| ***SLC3A2* (4F2hc) Chr:11** | | | | | |
| rs12794763 T/G | 62858040 | Intron variant | 0.04 | 0.15 |  |
| rs12221878 C/G | 62858559 | Intron variant | 0.09 | 0.07 |  |
| rs10792362 T/C | 62873879 | Intron variant | 0.41 | 0.43 |  |
| rs12804553 G/T | 62876155 | Intron variant | 0.29 | 0.28 |  |
| [rs489381](https://www.ensembl.org/Homo_sapiens/Variation/Explore?db=core;g=ENSG00000168003;r=11:62856004-62888880;v=rs489381;vdb=variation;vf=164693309) G/A | 62884591 | Intron variant | No HWE | 0.11 |  |
| rs4726 C/T | 62885307 | Synonymous variant | 0.24 | 0.25 |  |
| rs2282477 T/C | 62889032 | 3'near gene variant | 0.14 | 0.23 |  |

MAF: Minor Allele Frequency; HWE: Hardy Weinberg Equilibrium; TSI: Tuscany, Italy
