# Supplementary material for: Evidence for a relationship between genetic polymorphisms of the L-DOPA transporter LAT2/4F2hc and risk of hypertension in the context of chronic kidney disease
Source: BMC Med Genomics. 2024 Jun 18;17:163. doi: 10.1186/s12920-024-01935-2 (PMC11186288; doi:10.1186/s12920-024-01935-2)
Supplement: Supplementary file 3 — Supplementary Material 3 [file 12920_2024_1935_MOESM3_ESM.docx]

**Additional file 3**. Characteristics of the study participants

| **Variables** | **Whole sample**  (n= 421) |
| --- | --- |
| Age, years | 78.57 (9.64) |
| Gender, males % | 48.22 |
| Body mass index, kg/m^2^ | 26.44 (4.36) |
| Systolic blood pressure, mmHg | 135.92 (15.53) |
| Diastolic blood pressure, mmHg | 76.45 (8.52) |
| Triglycerides, mg/dl | 133.01 (71.88) |
| HDL-cholesterol, mg/dL | 55.44 (13.89) |
| LDL cholesterol, mg/dl | 123.10 (35.12) |
| Total cholesterol, mg/dl | 204.94 (42.35) |
| Fasting plasma glucose, mg/dL | 109.02 (36.30) |
| Glycated hemoglobin, % | 5.52 (1.01) |
| Albumin (g/dL) | 3.96 (0.36) |
| Total protein (g/dL) | 6.91 (0.50) |
| Creatinine (mg/dL) | 1.07 (0.38) |
| Urea (mg/dL) | 39.43 (16.41) |
| Uric acid (mg/dL) | 5.13 (1.50) |
| Sodium (mM/L) | 139.41 (2.55) |
| Potassium (mM/L) | 4.39 (0.44) |
| Chloride (mM/L) | 104.24 (2.46) |
| Calcium (mg/dL) | 9.34 (0.56) |
| Phosphorus (mg/dL) | 3.46 (0.73) |
| Magnesium (mg/dL) | 2.02 (0.29) |
| Iron (μg/dL) | 80.08 (37.77) |
| Ferritin (ng/mL) | 118.14 (154.97) |
| Total biluribin (mg/dL) | 0.74 (0.35) |
| Alkaline phosphatase (U/L) | 101.80 (65.16) |
| C-Reactive Protein (mg/L) | 5.83 (10.43) |

Variables are expressed as mean (standard deviation).
